# Primary Non-Hodgkins lymphoma of the parotid gland

**DOI:** 10.1590/S1808-86942011000500017

**Published:** 2015-10-22

**Authors:** Francesco Dispenza, Giuseppe Cicero, Gianluca Mortellaro, Donatella Marchese, Gautham Kulamarva, Carlo Dispenza

**Affiliations:** 1 MD (Research Doctor); 2 MD PhD (Professor); 3 MD (Consultant); 4 MD (Resident); 5 MS DNB DOHNS MRCS (Professor); 6 MD (Professor)

**Keywords:** lymphoma, lymphoma, non-hodgkin, parotid neoplasms, radiotherapy, salivary glands

## Abstract

**Abstract:**

Primary malignant lymphomas in the salivary glands are relatively rare. Clinical presentation is not characteristic and the disease is often overlooked resulting in diagnosis and treatment delays.

**Aim:**

To stress the importance of the diagnostic process and combined management, we present a series of eight patients with malignant lymphoma of the parotid who were diagnosed only after surgery and managed with radiation and chemotherapy.

**Methods:**

Retrospective series of patients with primary malignant lymphoma of the parotid gland managed with radiotherapy and diagnostic surgical partial resection.

**Results:**

After treatment completion we achieved a loco-regional control rate of 87.5%. Toxicity was charted according to the Common Toxicity Criteria and it was seen in six patients (75%). Six patients are still alive without evidence of recurrent disease in their last follow-up.

**Conclusion:**

Our study confirms that primary early stage Non-Hodgkin Lymphoma of the parotid gland is a disease with an excellent prognosis and a good local control rate, with minimal morbidity.

## INTRODUCTION

Among lymphatic tumors involving the head and neck, Non-Hodgkins lymphomas (NHLs) represent just 2%-4% of them. They represent 2%-6% of the tumor-related mortality amongst all the head and neck lymphomas. Its prevalence increases with age (peak incidence at 55 years of age) and the male/female ratio is 2:1. Generally surgeons do not anticipate primary NHL in the salivary glands pre-operatively and pathologists too find it difficult to give a definitive and diagnostic report based on either frozen section or fine needle aspiration biopsy (FNAB). This difficulty in pre and intra-operative diagnosis often results in unnecessary radical operations to the patient with all its associated risks involved.

The pathophysiology of NHL is unknown. However, either acquired or drug-induced immunodeficiency increases the risk by 50-100 times. Some autoimmune disorders are increasingly recognized as risk factors for NHL. In a recently conducted pooled analysis of 12 case-control studies including 29,423 participants, Sjogren's syndrome (SS) was found to be associated with a 1000 fold increase in the risk of parotid gland marginal zone lymphoma and a 6.5 fold increase in the risk of NHL overall^1^. Usually NHLs arise from B-lymphocytes with variable grade of differentiation and may involve lymph-nodes (nodal origin) or structures having abundant lymphatic tissue (extranodal origin).

Primary parotid gland lymphoma is a small subset of head and neck NHL. The overall incidence is about 0.3% of all tumors, 2%-5% of salivary gland neoplasm and 5% of extranodal lymphomas^2^, ^3^, ^4^. In contrast to other extranodal locations of NHL, parotid gland involvement is more likely to be of low grade and the patients have a better prognosis than do those with other extranodal NHL^5^.

The treatment of primary parotid lymphoma includes radiotherapy and chemotherapy as suggested in several reported series. Surgical excision is advocated only as a diagnostic tool.

We present our results in managing eight parotid gland lymphomas with combined radio-chemoterapy.

## MATERIAL AND METHODS

We have done a retrospective study of all the patients managed in our department for parotid gland surgery during a 20 years period from 1988-2008. The patients managed for NHL of the parotid gland were extracted and analyzed. Only patients who presented with a swelling of the parotid as their first symptom, who did not have a previous diagnosis of lymphoma and who later on were confirmed histologically post surgically as a parotid gland lymphoma were included in the study. The following patients were excluded from the study. 1) Patients who had lymphoma diagnosed in the past; 2) Patients who had simultaneous other salivary gland involvements; 3) Those patients who presented with other symptoms of lymphoma and later on diagnosed as having a parotid gland involvement.

History and clinical data were recorded from the clinical notes. All the patients had a complete head and neck examination. The instrumental evaluation included: Computed Tomography (CT) with contrast enhancement, fine needle aspiration biopsy (FNAB) and/or histological analysis on a specimen surgically obtained. In all the cases a superficial parotidectomy with preservation of the facial nerve was done after consultation with the patients. All the surgically obtained specimens underwent a definitive histopathologic examination. All patients were staged according to the Ann-Arbor staging system (Table 1). Staging consisted of a general physical examination, hematological screening, CT of chest, abdomen and pelvic regions and bone marrow biopsy. Histopathology of the specimen was described in accordance with Updated Kiel classification. After diagnosis, all the cases received radiation-therapy (RT) and chemotherapy (ChT).

**Table 1:** Ann Arbor Staging System for Non-Hodgkin's Lymphoma.

Stage	Description
I	Involvement of a single lymph node region or of a single extranodal organ or site (IE).
II	Involvement of two or more lymph node regions on the same side of the diaphragm, or localized involvement of an extranodal site or organ (IIE) and one or more lymph node regions on the same side of the diaphragm.
III	Involvement of lymph node regions on both side of the diaphragm, which may also be accompanied by localized involvement of an extranodal organ or site (IIIE) of spleen (IIIS) or both (IIISE).
IV	Diffuse or disseminated involvement of one or more distant extranodal sites.

The patients received RT as conformal external beam radiation to the parotid bed and also neck lymph-nodes levels I-II & III, when indicated by the stage of the disease. Treatment was delivered in all cases using unilateral anterior and posterior wedged-pair fields using 6 MV photons, MLC (multi leaf collimator) collimated. The target volume was expanded by one cm set up margin to generate the planning target volume (PTV). We also boosted the RT with the help of electron beam of 9/12 MeV in six patients who had superficial extension of the lesion. With the wedged-pair technique, slight inferior angulations of the beams avoided an exit dose to the contralateral eye.

Only patients with a minimum follow-up of one year were included. Our institutional review board approved the study.

## RESULTS

Among 346 patients undergoing parotid gland surgery during the last 20 years, 11 patients had a diagnosis of parotid gland NHL. The first patient with diagnosis of NHL was recorded in 1995. The mean age was 60 years (range 45-80 years). Male/female ratio was 2:1. Three out of 11 NHL patients were excluded from the retrospective review as they had a previous diagnosis of lymphoma within the past three years.

All eight patients complained of a parotid gland swelling and in two patients there was bilateral enlargement of the parotid. Head and neck examination did not reveal any neck node enlargement in any of the patients. Imaging studies showed, in all cases, a well demarcated increase in the volume of the parotid without any signs of enhancement or invasion of the surrounding tissue. The FNAB, obtained in seven patients, were all negatives for malignant lymphatic lesion. In two cases it was indicative of Warthin's tumor, in another two cases it suggested a benign lymphoepithelial lesion; and in the remaining three cases it only gave an inconclusive result. One patient did not have an FNAB done. The diagnosis of NHL in all patients was established after definitive histopathologic examination of the resected surgical specimen. In two patients with bilateral swelling the operation was done on the side with bigger mass for patients discomfort and with the purpose of definitive diagnosis. Five patients had follicular B cell lymphoma with predominantly small-cleaved cell and the remaining three had a diagnosis of diffuse large cell lymphoma. After staging tests: five patients were in stage I and three patients were in stage II.

After diagnosis, all patients were treated with RT and ChT with CHOP or R-CHOP protocols administered at an interval of two weeks (cyclophosphamide 750 mg/ m2, doxorubicin 50 mg/m2, vincristine 1.4 mg/m2, prednisone 100 mg for 5 days every 14 days). Rituximab was administered in four cases diagnosed after 2006, but no differences in outcomes were noted, probably due to low number of cases. Patients with NHL stage I underwent three cycles of chemotherapy, while those with stage II had three additional cycles. All patients received conformal external beam RT to the parotid bed, the three patients with stage II disease received irradiation to the neck up to level III group of lymph nodes. Median dose delivered was 38,4 Gy (range 36,8-40 Gy) at 1.8 to 2.0 Gy per fraction. Tolerance of the patient to the radiation dosage decided the final RT dosage delivered.

Median follow-up was two years (range one to four years). After RT a regional and local control, defined as no evidence of recurrent disease within the head and neck and the treated fields respectively, was obtained in 7/8 patients (87.5%), one patient (12.5%) in which no electron beam boost was given, presented with local recurrence after six months. Toxicity was retrospectively scored according to the Common Toxicity Criteria v. 2.0 (CTC v. 2.0)^6^: six patients (75%) had grade I mucositis and two patients grade I dysphagia. (Table 2 and 3) None of the seven recurrence free patients had palpable or detectable residual disease in the last follow-up. Six patients are surviving and are disease free at their last follow-up. One patient with recurrent disease died after eight months from recurrence. One patient died of other causes not related to the lymphatic disease. He died after two years following his treatment and was disease free at his last follow up.

**Table 2:** Dysphagia pharyngeal related to radiation CTC v. 2.0.

Grade	Description
0	None.
1	Mild dysphagia, but can eat regular diet.
2	Dysphagia, requiring predominantly pureed, soft, or liquid diet.
3	Dysphagia, requiring feeding tube, IV hydration or hyperalimentation.
4	Complete obstruction (cannot swallow saliva); ulceration with bleeding not induced by minor trauma or abrasion or perforation.

**Table 3:** Mucositis due to radiation CTC v. 2.0.

Grade	Description
0	None
1	erythema of the mucosa
2	patchy pseudomembranous reaction (patches generally <1.5 cm in diameter and noncontiguous)
3	confluent pseudomembranous reaction (contiguous patches generally >1.5 cm in diameter)
4	necrosis or deep ulceration; may include bleeding not induced by minor trauma or abrasion

## DISCUSSION

Majority of extranodal lymphomas of the head and neck are NHL and in 4%-5% of cases the parotid gland is involved^3^,^7^. Primary parotid gland NHL represents 1%-4% of all parotid tumors. These numbers, however, cannot indicate the true prevalence of the disease^8^, ^9^, ^10^. The true prevalence of such disease is not quite obtained because of poor consistency of the series available. Reports present in the literature are old and in the last decade there were few series describing such disease.

Another cause of confusion in literature about the true incidence and then the real prognosis is the inadequate staging. Such lack of patients examination data is more evident in the earlier cases described due to the limited availability of imaging and technology at that point of time which in today's day and age is considered as obsolete. As reported by Lipton et al in 1971, as many as 35% of patients presenting with NHL apparently confined to the head and neck region were found to have intra-abdominal disease after adequate staging^11^.

There is a debate in the scientific literature about the true origin of lymphomas within the parotid gland. Batsakis assessed that the propensity of parotid gland to be involved by NHL is related to the anatomy of the gland, rich in lymph nodes and lymphoid tissue^12^. Non-Hodgkins lymphoma of the parotid gland may be classified as extranodal if the origin is from the mucosa associated lymphoid tissue (MALT) or nodal if the true origin is from lymph node within the gland. The lesions, in the opinion of some authors, arise from the lymph nodes associated with the gland and then the gland tissue around the lesion is involved secondarily. This theory makes it difficult to establish the true origin of the disease^12^.

There are also other workers in the field who suggests that, salivary glands do not normally contain MALT but may acquire it as a result of an autoimmune inflammatory disorder like Sjogren's syndrome (SS)^13^. However, the true origin of NHL in the parotid gland is actually without much relevance because the treatment, till now, is not influenced by such detail^10^. We too believe in this viewpoint. It is generally considered that the great number of cases presenting with a stage I disease have a MALT disease and the clinical course is indolent requiring a less aggressive treatment^14^. In our current series though, we didn't find MALT lymphoma patients, eventhough we had cases identified as stage I disease. None of our patients had SS either again supporting the suggestion that MALT lymphoma is more likely to be seen in patients with SS.

Although considered insufficient by some authors^15^, Hyman & Wolff^16^ have suggested some criteria to consider a lesion as primary parotid gland lymphoma: 1. Involvement of the gland should be the first clinical manifestation of the disease; 2. Histologically, the disease should involve the gland parenchyma and not adjacent lymph nodes or soft tissue alone; 3. There should be confirmation of the malignant nature of the lymphoid infiltrate. We adopted these criteria to consider as primary NHL a lymphatic malignancy involving the parotid glands.

Diagnosis of parotid lymphoma is often not easy when a new parotid gland mass is evaluated. Clinical presentation is indistinguishable from other benign parotid swellings, but there is some clinical and historical features that should make the physician consider lymphoma as possible etiology of the lump.

In our series, as well as in the literature examined, the mean age at diagnosis is around 60 years of age. Males were affected more frequently than females for which the reason is not clear. Incidence of an underlying autoimmune disorder in such patients has been reported to range from 0% to 44%^16^,^17^. Therefore we should have a high index of suspicion in such patients when presenting with parotid lumps. A cervical lymphoadenopathy also should make one consider this diagnosis^18^.

Most primary salivary gland lymphomas are B marginal zone lymphomas arising on a background of sialadenitis associated with autoimmune disorders such as Sjogrens syndrome. Primary T-cell lymphoma of the salivary gland is rare^19^. In our series up to five of our patients belonged to the follicular B cell variant and the remaining three were of large cell lymphoma type. However none were observed to be associated with autoimmune disorders.

It was noted that autoimmune disorders affecting salivary glands may predispose to uncontrolled proliferation of lymphatic tissue^10^,^12^,^20^. In these patents NHL consisted of a monoclonal proliferation of B-cell, which arose in the polyclonal infiltrates typical of benign lymphoepithelial lesion that we can also find in Sjogren's syndrome^21^. Sjogren's syndrome is associated with 6.5 fold increase in the risk of NHL, 250 fold increase in the risk of parotid gland NHL and a dramatic 1000 fold increase in the risk of parotid gland MALT lymphoma. Biologically, it is well documented that, antigen driven immune stimulation develops MALT lymphoma in parotid gland in Sjögren's syndrome^1^.

There is a difficult group of patients with or without Sjögren's syndrome that develop atypical lymphoreticular proliferation into salivary glands. Such lesion is between lymphoreticular hyperplasia and neoplasia, known as pseudolymphoma^22^. This variety of lesions and histological pictures may explicate the varied opinion that either doubt FNAB or frozen section and are often followed or substituted by superficial parotidectomy in order to reach the definitive diagnosis. The coexistence of Sjögren's syndrome gives such patients a worse prognosis^23^. Survival period in this group of patients is generally less than three years. This reduction of the survival period could be due to both frequent systemic involvement (lacrymal, digestive, cutaneous localization) and the high incidence of worse histological type of NHL in these patients. This thought is confirmed on observing the survival rate of Sjogren's patients with isolated low grade NHL, who have the same survival rate as of other patients with parotid gland NHL disease but without SS^12^,^14^,^24^.

Warthin's tumour and malignant lymphomas are only rarely assossiated. Follicular lymphoma is the most common lymphoma presenting as a collision tumour with Warthin's^25^. Eventhough we had two diagnosis of Warthin's in the preoperative FNAB, none were confirmed in the final histology. This was even when we had five follicular lymphomas confirmed histologically.

Freedman et al.^7^ noted a relationship between histopathology and survival^26^; nodular type was associated with prolonged survival in his series. In the present small group of patients survival was not related to either histological grade or pattern of lymphoma.

Reported rate of systemic dissemination of NHL as available in the current literature is variable between 30% to 60%^3^,^16^,^27^. The relative low rate of advanced stage (III-IV) reported in the parotid gland NHL series is contradictory with the general findings in NHL of other region, where the majority of patients have diffuse disease at the time of diagnosis. Our series showed the same trend of stage at presentation where all the patients belonged to either stage I or II.

Survival rate for parotid lymphoma vary considerably from report to report and is generally better than other extranodal lymphomas in which the survival rate is not over 41%^7^. The majority of authors reported a longtime survival rate ranging from 50% to 75%^3^,^7^,^26^, ^27^, ^28^, ^29^. There is consensus between authors, regarding the grade and the histology of the lymphoma, that they do not appear to consistently modify the prognosis^30^. Interestingly it appears that, there can be a relation between the tumor size and survival. It was noted that if the parotid lesion has a diameter of less than six centimeter of diameter, the five years survival rate reaches 87%, on the other hand when the greatest diameter is more than six cm the rate decreases to 61%^31^,^32^. In any case, surgical reduction of the tumor by superficial parotidectomy is not related to better prognosis^30^.

Patients with stage I intermediate to high-grade NHL or stage II low-grade disease can benefit from RT alone^8^,^9^,^28^,^30^. The local control rate reached with RT in management of lymphomas is very high as reported by several studies^33^,^34^. In our series we obtained a complete local control in 87% of patients with chemotherapy and radiotherapy. The fact that no palpable lesion was present in the follow-up is noticeable because of the strict relationship between tumor volume and survival as mentioned before.

Chemotherapy is considered as an useful adjunctive treatment in these patients^8^,^9^ and it was used in all our cases. Patients with advanced stage (II or III) should be treated with chemotherapy if the disease is curable or if the patients are symptomatic. In case of low grade pattern or if the patients are asymptomatic, chemotherapy may be delayed as assessed by some authors^35^.

The complication rate for patients we managed was relatively low with six (75%) cases of grade I mucositis and two (25%) cases of associated transitory dysphagia.

The only role for surgery in these cases is as a diagnostic tool. We recommend, in agreement with several other authors, a superficial parotidectomy for all undiagnosed lumps of the gland and for unclear FNAB results^3^,^18^,^36^, ^37^, ^38^, ^39^. Upon receiving a positive frozen section diagnosis of parotid lymphoma, the procedure should be stopped immediately and no further excision should be performed.

## CONCLUSIONS

Presence of a parotid gland swelling is a common presentation in the clinical practice and patients affected by lymphomas are clinically indistinguishable from those having a benign lesion. Presence of history of autoimmune disease and a clinically appreciable lymphoadenopathy in these patients should be raising the index of suspicion for a lymphatic malignancy. The diagnostic pathway must include imaging and a FNAB that may be diagnostic in some cases.

Majority of the patients however, require a superficial parotidectomy to reach a definitive diagnosis, because both FNAB and frozen section are often inconclusive. A complete assessment and staging is mandatory before commencing therapy. Patients who present with an early stage or low-grade primary parotid gland NHL have a better prognosis. Although the number of cases in our series is small and the follow-up is limited, the treatment protocol including RT and chemotherapy showed in our series a good response rate with minimal recurrence risk, giving the physician and patients the much needed reassurance in its use.
